# High metallothionein predicts poor survival in glioblastoma multiforme

**DOI:** 10.1186/s12920-015-0137-6

**Published:** 2015-10-22

**Authors:** Ruty Mehrian-Shai, Michal Yalon, Amos J. Simon, Eran Eyal, Tatyana Pismenyuk, Itai Moshe, Shlomi Constantini, Amos Toren

**Affiliations:** Pediatric Hemato-Oncology, Edmond and Lilly Safra Children’s Hospital and Cancer Research Center, Sheba Medical Center, Tel Hashomer affiliated to the Sackler School of Medicine, Tel-Aviv University, Tel Aviv, Israel; Department of Pediatric Neurosurgery, Dana Children’s Hospital, Tel-Aviv-Sourasky Medical Center, Tel-Aviv, Israel

**Keywords:** Glioblastoma multiforme, Survival, Metallothionein, Zinc, *p53*

## Abstract

**Background:**

Glioblastoma multiforme (GBM) is the most common and aggressive malignant brain tumor. Even with vigorous surgery, radiation and chemotherapy treatment, survival rates of GBM are very poor and predictive markers for prognosis are currently lacking.

**Methods:**

We performed whole genome expression studies of 67 fresh frozen untreated GBM tumors and validated results by 210 GBM samples’ expression data from The Cancer Genome Atlas.

**Results and discussion:**

Here we show that in GBM patients, high metallothionein *(MT)* expression is associated with poor survival whereas low *MT* levels correspond to good prognosis. Furthermore we show that in U87 GBM cell line, *p53* is found to be in an inactive mutant-like conformation concurrently with more than 4 times higher *MT3* expression level than normal astrocytes and U251GBM cell line. We then show that U87*-* p53 inactivity can be rescued by zinc (Zn).

**Conclusions:**

Taken together, these data suggest that *MT* expression may be a potential novel prognostic biomarker for GBM, and that U87 cells may be a good model for patients with non active WT *p53* resulting from high levels of *MT*s.

## Background

Glioblastoma multiforme (GBM) is the most common and aggressive malignant brain tumor [1]. Survival rates vary between studies (20.0 to 61.1 % at 1 year) and only 2 %–5 % of patients are expected to survive longer than 2 years [2]. Our understanding of these tumors has expanded recently by efforts made by The Cancer Genome Atlas (TCGA) and others [3]. These efforts have led to the discovery of new molecular biomarkers for disease prognosis including microRNAs dysregulation [4], methyl guanine methyl transferase (*MGMT*) promoter methylation [5] and mutations in isocitrate dehydrogenase 1 (*IDH1*), *EGFR*, and *p53* [6]. Nevertheless, the predictive ability of outcome by imaging, clinical, and genomic biomarkers is still only 69 % [7] and accordingly new biomarkers are needed.

**Table 1:** List of Affymetrix probes used for *MT*s and *HIPK2*

Number	Gene	Probe
1	*MT1X*	204326_x_at
2	*MT1G*	204745_x_at
3	*MT3*	205970_at
4	*MT1H*	206461_x_at
5	*MT1X*	208581_x_at
6	*MT1P2*	211456_x_at
7	*MT2A*	212185_x_at
8	*MT1E*	212859_x_at
9	*MT1F*	213629_x_at
10	*MT1M*	216336_x_at
11	*MT1F*	217165_x_at
12	*MT1M*	217546_at
13	*HIPK2*	213763_at
14	*HIPK2*	219028_at

Metallothioneins (*MT*s) are intracellular heavy metal binding proteins. Mammalian *MT*s fall into 4 subgroups: *MT1, MT2A, MT3, and MT4* [8]. *MT1* encodes multiple isoforms, *MT1A*, *MT1B, MT1E, MT1F, MT1G, MT1H, and MT1X* [9]. *MT1* and *MT2* are expressed in nearly all organs [9]. *MT3* is preferentially expressed in the brain, while *MT4* expression appears to be limited to squamous epithelial cells [10, 11]. *MT*s can bind 7 atoms of zinc per molecule of protein [12] and affect the cellular zinc amount [13]. They also bind cytotoxic agents, such as alkylating agents and confer resistance to these anticancer drugs. *MT*s over expression has been linked to chemo resistance in carcinoma cells, non-small cell lung cancer cell lines, tongue squamous cell carcinoma cell lines, gastric tumor cell lines, ovarian carcinoma cell lines and osteosarcoma [14]. Elevated levels of *MT1X, MT1F, MT2A , MT1A, MT1E* and *MT3* were shown in result of arsenic trioxide chemotherapy drug on U87 cells by Falnoga et al and postulated as potential mechanisms for GBM resistance [15]. Furthermore, it has been shown that the metal-free form of *MT* (apo-*MT*) may also bind P53 and prevent its activity [16–18]. Elevated levels of *MT1E* has also been shown to enhance the migration and invasion of human glioma cells [19].

*MT*s are negatively regulated by *HIPK2* [13]. It was previously reported that, in *HIPK2* knockdown colon cancer, breast cancer and ovarian cancer, p53 undergoes misfolding which inhibits DNA binding and transcriptional activities concurrently with increased chemoresistance [20, 21]. In MCF7 breast cancer cells, *HIPK2* knockdown is correlated with metallothionein 2A (*MT2A*) up regulation. Inhibition of *MT2A* expression in these cells by siRNA restores p53 transcription activity and drug-induced apoptosis [13]. Since p53 activity is zinc dependent, zinc deficiency may also halt p53 activity [20]. Absence of p53 activity, or expression of mutant *p53* (mtp53) are common in human cancers and are associated with increased cancer resistance to chemo- and radiotherapy. Therefore, significant efforts towards pharmaceutical reactivation of defective *p53* pathways are underway (reviewed in [22]). Zinc, for example, has been suggested as it re-establishes chemo sensitivity in breast cancer SKBR3 (expressing R175H mutation) and glioblastoma U373MG (expressing R273H mutation) cell lines to adriamycin and cisplatin treatment, respectively [23].

The Cancer Genome Atlas (TCGA) study showed that *p53* and its pathway are altered in 78 % of GBMs [24]. *p53* hotspot mutations include disruption of DNA interaction and DNA binding interface structure stabilization, which can be restored to wild-type configuration by *p53* reactivating treatments [25, 26]. For example, the R175H mutant induces structural distortions in *p53* protein that prevent it from binding zinc [27]. It has been shown that NSC319726 compound restores *p53* (R175) mutant structure to wild type conformation by its zinc ion metallochaperone properties [28].

In this study we report that in GBM patients, high metallothionein *(MT)* expression is associated with poor survival whereas low *MT* levels correspond to good prognosis. Furthermore we show that in U87 GBM cell line may be a good model for patients with non active WT *p53* resulting from high levels of *MT*s.

## Methods

Human subjects and cell lines: 67 fresh frozen untreated GBM tumors and seven postmortem normal brain samples (subcortical white matter) from UCLA tumor bank were used for the discovery set. Tissues were obtained at the time of surgery in accordance with applicable human ethics regulations approved by UCLA institutional review board and after written informed consent were obtained. Subjects were chosen to have similar condition and treatment. Patients underwent maximal tumor resection, received external beam regional radiation of 60 Gy within 3–6 weeks after resection, concurrent with temozolomide (75 mg per square meter of body-surface area per day, 7 days per week from the first to the last day of radiotherapy), followed by six cycles of adjuvant temozolomide (150 to 200 mg per square meter for 5 days during each 28-day cycle). The median age at diagnosis was 53 years and Karnofsky performance status (KPS) > 60. No patient died from disease or other causes not related to their brain tumor. The samples were snap frozen in liquid nitrogen. In addition data from 210 TCGA GBM samples were collected. The results shown here are based upon data generated by the TCGA Research Network: http://cancergenome.nih.gov/. KPS was high in the TCGA data set with a median value of 90. The median age at diagnosis for TCGA samples was 57 years and there were 62 % males. The human glioblastoma cell lines U87-MG (with wild type p53) obtained from the American Type Culture Collection (ATCC) and U251-MG (bearing mutant p53) were developed in the laboratory of Dr. J. Ponten, University of Uppsala, Sweden. Normal astrocytes were a kind gift of Dr. Chaya Brodie lab, Bar-Ilan Institute, Israel.

Microarray analysis: For the 67 discovery set, U95Av2 (Affymetrix, Santa Clara, CA) was used to interrogate 12,533 probe sets encoding 10,000 genes. Gene expression profiling studies of these samples were generated according to standard Affymetrix protocols. DNA-Chip Analyzer (dChip) (http://www.hsph.harvard.edu/cli/complab/dchip/) program was used to obtain model based gene expression value (measures the fluorescence intensity of that gene), then the class neighbors analysis standardizes the expression values for each gene by linearly adjusting their values across all samples to a mean of zero with a standard deviation of one. Individual genes are then clustered using an algorithm in dChip program that determines the correlation coefficients (r values) for the normalized expression values (distances between genes are defined as 1 – r). Genes with the shortest distances between them are grouped. The color scale for the resulting picture: the red color represents expression level above mean expression of a gene across all samples, the black color represents mean expression and the green color represents expression lower than the mean. Since the expression levels for each gene is standardized to have mean 0 and standard deviation 1, the standardized expression values fall within [-3, 3]. Thus in the resulting picture, black represents 0, pure red represents 3 or higher and pure green represents -3 or lower expression. After the discovery of genes that are predictive of survival, their mean expression value were plotted and compared between samples. In the clustering step the comparison is between the patients according to the survival time. The value for normal sample expression is plotted on the expression plot for base line orientation.

For the 210 TCGA GBM samples Affymetrix HT Human Genome U133 expression data were loaded from TCGA data portal (https://tcga-data.nci.nih.gov/tcga). Table 1 enlists Affymetrix probes used for MTs and HIPK2 analysis. The TCGA Data Portal application, Data Browser, was used to generate the Kaplan Meier curves.

Quantitative real time PCR: qRT-PCR experiments of human-derived GBM cell lines U251, U87 and normal human astrocytes were performed. U251 and U87 Cell lines were cultured in Dulbecco’s modified Eagle’s medium (DMEM; Gibco) supplemented with 10 % fetal bovine serum and 1 % penicillin/streptomycin. Normal human astrocytes (NHA) served as controls and were cultured in Astrocyte Basal Medium (ABM; Lonza) supplemented with 0.1 % rhEGF, 0.25 % Insulin, 0.25 % Ascorbic Acid, 0.1 % GA-1000, 1 % L-Glutamine, 3 % FBS and 1 % penicillin/streptomycin. All of the cell lines were cultured at 37 °C, in a 5 % CO2 atmosphere. Total RNA was extracted from 5x105 cells using the RNeasy Mini Kit (Qiagen, USA) following the manufacturer’s instructions. For quantization and quality control we used the Agilent 2100 Bioanalyzer (Agilent Thechnologies). A260/A280 Ratio of the RNA was approximately 2 for all samples. 2 ng RNA was used for reverse transcription by the High-Capacity cDNA Reverse Transcription Kit (Applied Biosystems - Life Technologies USA). The resulting 2 ng cDNA was used for the SYBR Green reagent-based RTPCR on Applied Biosystems 7500 Real Time PCR System. The primers that were used for MT3 RTPCR were: forward primer-5’ACCTCCTGCAAGAAGAGCTG3’, reverse primer- 5’C AGCTGCACTTCTCTGCTTCT3’ For β-actin forward primer-5’ CCTGGCACCCAGCACAAT 3’, reverse primer- 5’ GCCGATCCACACGGAGTACT3’. PCR program: 95 °C for 10 min, 40 cycles of {95 °C for 15 s and 62 °C for 1 min}. The experiments were carried out in triplicates for each data point. The relative gene expression was calculated for the gene of interest using the ΔΔCT method, in which cycle threshold (CT) values were normalized to the beta actin reference gene.

Immunofluorescent studies: For immunofluorescent staining of p53, cells were grown on coverslips, fixed with 4 % paraformaldehyde, permeabilized with 0.5 % Triton X-100 and stained overnight with the PAb1620 (WT conformation) (Calbiochem) or PAb240 (mutant conformation) (Calbiochem) antibodies and then with FITC-labeled (green) secondary antibody, goat antimouse IgG (millipore). DAPI fluoromount (blue) (Southern Biotech) was used for nuclei labeling. The cells were examined by fluorescence microscopy.

Quantitative measurements of the fluorescent signal: Staining intensity of a fluorescent PAb240 and PAb1620 antibody were recorded using an indirect method. The digitized images were processed to determine quantitative measure for the specific fluorescence signal (the background and nuclear blue corrected fluorescence in the object) using existing tools in the widely used image processing software Photoshop from Adobe. The region of interest was defined in the TIFF format image, where the 3 RGB channels, blue, red and green are encoded as separate layers. Using the histogram function in Photoshop we determined the intensity of the light (fluorescence) for that individual color channel.

Cell treatment: U251 and U87 cell lines were plated in triplicates for 24 h in order to allow adherence. ZnCl_2_ (Sigma-Aldrich) was added at final concentration of 100 μM.

## Results

**Fig. 1:**
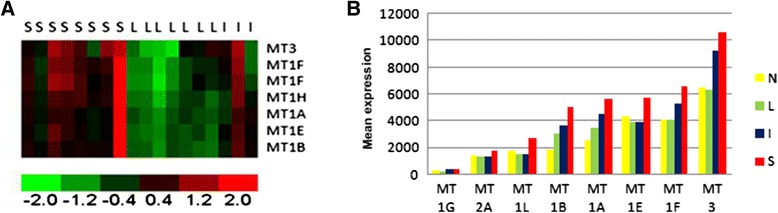
**a** Illustration of different *MT*s expression in 15 untreated patients with short (S) Intermidiate (I) and long (L) survival – a ‘heat map’. Largest relative gene expression values are displayed in red, the smallest relative values in green and intermediate values in shades of black. **b** Mean expression values of different *MT*s in 67 untreated patients with short (S) long (L) and intermediate (I) survival and 7 (N) normal brain

We analyzed whole genome expression data of a discovery set of 67 untreated tumors from GBM patients to find novel survival biomarkers. Patients were grouped to short (S) (less than 300 days), long (L) (more than 1095 days- 3 years) and intermediate (I) (between 300 to 1095 days) survival. First, to get the most effective survival markers intermediate survival samples were eliminated. dChip class neighbors analysis revealed that a group of metallothionein genes clustered the 8 short survival and 7 long survival samples. Expression of Metallothionein-3, 1 F, 1H, 1A, 1E and 1B were relatively much higher in short survival patients compared to long survival patients (Fig. 1a). Mean RNA expression comparison of these MTs in all the samples and in normal brain tissue revealed that MT expression in long survival patients is similar to normal brain whereas in intermediate and short survival the expression is higher (Fig. 1b). *MT3* and *MT1f* showed the most significant difference between long and short survival patients (*p*-value 0.003 for both), thereafter, *MT1B* and *MT1A*, both with *p*-value 0.011 and finally *MT1G p*-value 0.012. Since there is a high positive correlation between *MT3* and *MT1F* expression (r = 0.87 *p* = 0.004E^−13^) we continued our study with *MT3*.

**Fig. 2:**
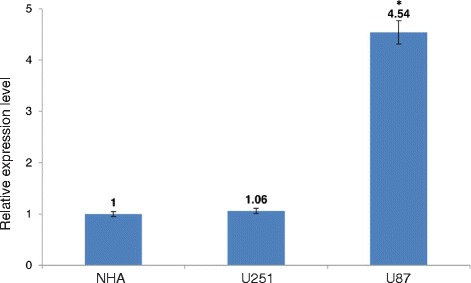
*MT3* expression in U251 and U87 GBM cell lines relative to normal astrocytes. Relative quantification (RQ) is reported as means ± SD of triplicates and statistical significance was determined by one-way ANOVA and post hoc Tukey’s test. *- *P* < 0.05 compared to NHA and to U251

As indicated, high *MT* expression may lead to an inactive p53 which is crucial in alkylating agent induced apoptosis. We looked for a cellular model for patients that have WT p53 but due to high levels of *MT*s, the misfolded conformation turns WTp53 to an inactive form. Quantification of *MT3* expression in normal astrocytes, U251 and U87 glioma cell lines revealed that *MT3* is elevated more than 4 fold in U87 cells (Fig. 2), compared to normal astrocytes.

**Fig. 3:**
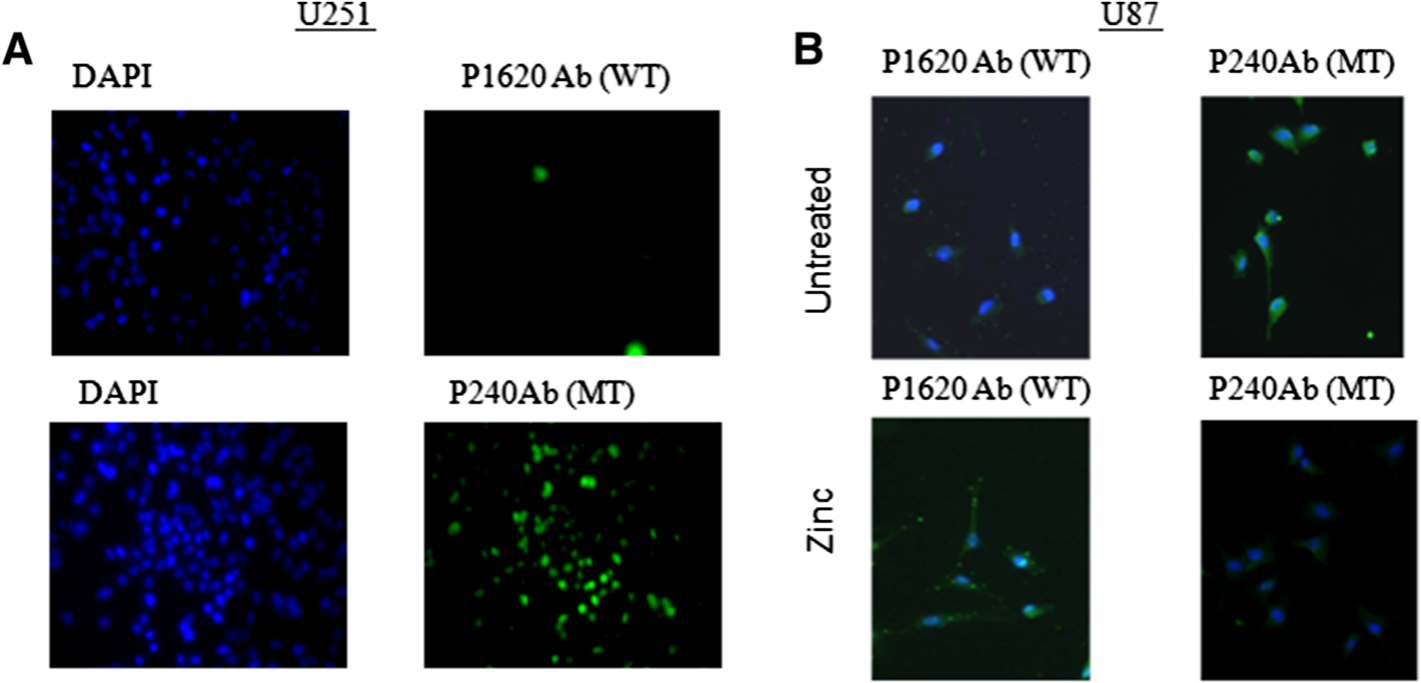
**a** p53 conformation is inactive in U251 cell line. Fluorescent microscope image with 4× objective of U251 cells stained with DAPI for nuclei (blue) and FITC (green) Ab for wild type (WT) *p53* (PAb1620) or FITC Ab for Mutant (MT) p53 (PAb240). **b** *p53* conformation in U87 cell line changes to active form upon zinc addition. Untreated /zinc treated superimposed fluorescent microscope image with 10× objective of U87 stained with DAPI for nuclei (blue) and FITC (green) Ab for wild type (WT) *p53* (PAb1620) or FITC Ab for Mutant (MT) *p53* (PAb240). After zinc supplementation in U87 cells, p53 conformation changed from inactive mutant like to active WT like

**Fig. 4:**
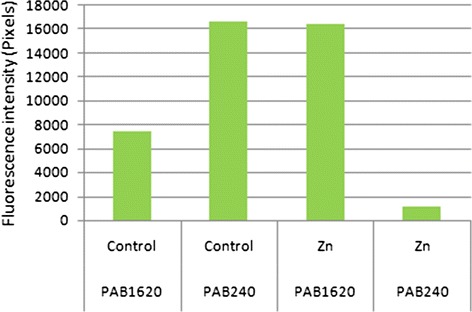
P53 conformation quantification in U87 before (control) and after addition of zinc (Zn). P53 conformation detected by FITC labeled (green) secondary antibody to PAb240 (mutant like conformation) and PAb1620 (wild type conformation), intensity (pixels) per cell is plotted for each conformation with and without zinc addition

We checked the p53 conformation in the U251 and U87cell lines by immunofluorecent labeling with the conformation-specific antibodies PAb1620 (for p53 wild-type, folded conformation) and PAb240 (for p53 mutant unfolded conformation). U251 harbors R273H mutation (The Tp53 mutation web site http://p53.free.fr/Database/Cancer_cell_lines/p53_cell_lines.html) and indeed these cells showed *p53* inactive conformation (Fig. 3a). U87 on the other hand is known to harbor wild type *p53* (The Tp53 mutation web site http://p53.free.fr/Database/Cancer_cell_lines/p53_cell_lines.html). Surprisingly, however, immunofluorescent staining with the specific WT (PAb1620) and mutant (PAb240) antibodies, respectively, revealed that most of the U87 *p53* is in a mutant conformation. Thus, although *p53* is not mutated in this cell line its conformation is inactive. As zinc can restore *p53* function by changing the conformation from a non active to an active conformation [23], we tested whether addition of zinc can rescue *p53* conformation in U87 cells. Indeed, zinc supplementation decreased mutant conformation and increased *p53* wild type conformation phenotype in U87 (Fig. 3b). Quantitative measurements of the fluorescent signal intensity of a fluorescent PAb240 and PAb1620 antibody confirmed the increased *p53* wild type conformation phenotype upon addition of zinc (Zn) (Fig. 4).

**Fig. 5:**
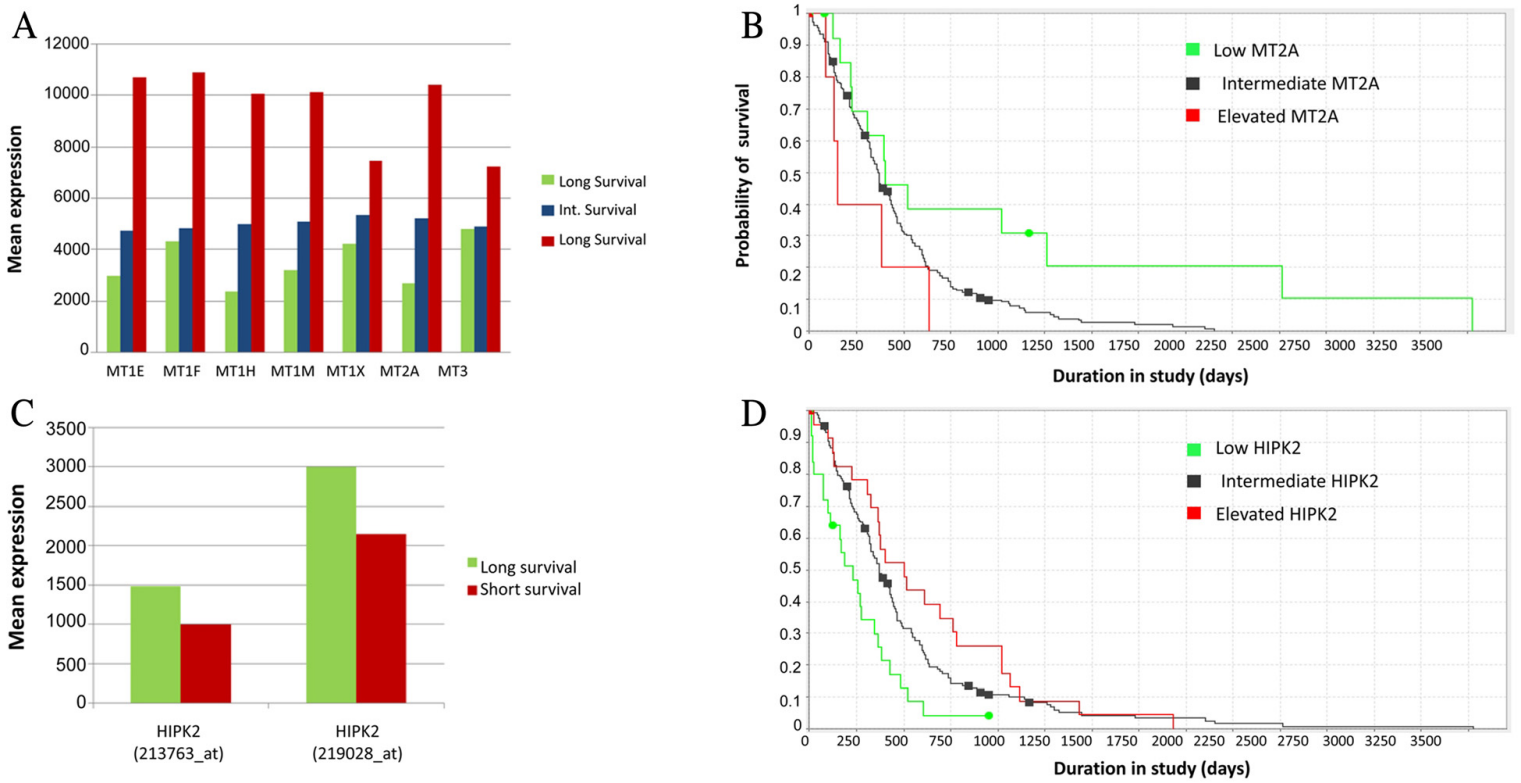
**a** *MT*s mean expression in short, intermediate (Int) and long survival GBM patients. M1E, Mt1F, MT1H, MT1M, MT1X, MT2A and MT3 mean expression intensity is higher in short survival patients compared to long survival patients. **b** Kaplan–Meier survival curve for *MT2A* expression. Red line- up regulated *MT2A*; green line- down regulated *MT2A*; black line - intermediate expression. **c** *HIPK2* mean expression intensity and survival in GBM samples. *HIPK2* Affymetrix probes (213763_at in red and 219028_at in blue). *HIPK2* is higher in high survival patients compared to low survival patients in both probe sets. **d** Kaplan–Meier survival curve for *HIPK2* expression. Red line- up regulated *HIPK2*; green line- down regulated *HIPK2*; black line- intermediate expression

**Fig. 6:**
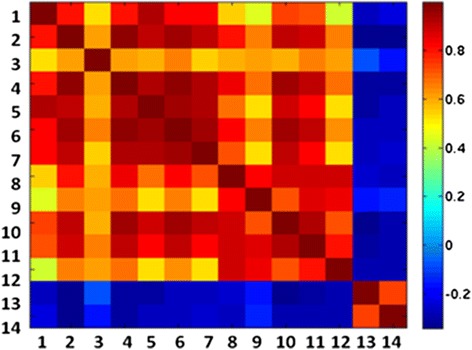
Heat map correlation coefficients between 12 probes of MTs. *MT1X, MT1G, MT3, MT1H, MT1X, MT1P2, MT2A, MT1E, MT1F, MT1M, MT1F, MT1M* (#1-12) expression and two probes (#13, 14) of *HIPK2* expression (Table 1). Red color represents highly positive correlation of expression and blue represents negative correlation. All MTs are highly positive (red) correlated to each other and negatively correlated (blue) to *HIPK2* probe

Next we checked the expression of *MT*s in 210 samples from The Cancer Genome Atlas (TCGA). We used TCGA portal, cBio Cancer Genomics Portal (http://cbioportal.org/). Table 1 enlists Affymetrix probes used for MTs and HIPK2 analysis. There were 71 samples with short survival, 113 with intermediate survival and 22 with long survival. Four samples were excluded because of missing data of either survival time or *MT* expression. All *MT*s that were quantified in this study had significantly higher mean expression intensity in short survival patients compared to long survival patients (Fig. 5a) (3.6 times for *MT1E*, 2.5 for *MT1F*, 4.2 times for *MT1H*, 3.2 times for *MT1M*, 1.8 times for *MT1x*, 3.9 times for *MT2A* and 1.5 times for *MT3*). *MT1G* expression intensity was much higher than all others, yet its expression is 1.8 times higher in short survival patients compared to long survival patients. Kaplan–Meier survival curve overlapping TCGA study core samples also illustrated shorter survival correlated to high *MT* expression. For *MT2A* (Fig. 5b) among the 210 samples there were 5 up-regulated samples, 14 down-regulated samples and 190 intermediary samples**.** The significance of difference in survival between groups of samples was calculated by log-rank *p*-value. Log-rank *p*-value of up-regulated vs. down-regulated is 0.054. Log-rank *p*-value of up-regulated vs. intermediary is 0.253 and log-rank *p*-value of down-regulated versus intermediary is 0.0139. Similar results were inspected for *MT1f*: 21 up-regulated samples; 22 down-regulated samples and 167 intermediary samples**.** Log-rank *p*-value of up-regulated vs. down-regulated is 0.0132. Log-rank *p*-value of up-regulated vs. intermediary is 0.23 and log-rank *p*-value of down-regulated vs. intermediary is 0.0145. For *MT1a* the results were not significant: 40 up-regulated samples, 50 down-regulated samples and 121 intermediary samples**.** Log-rank *p*-value of up-regulated vs. down-regulated is 0.196. Log-rank *p*-value of up-regulated versus intermediary is 0.27 and log-rank *p*-value of down-regulated versus intermediary is 0.07. For *MT3* there were 46 up-regulated samples, 33 down-regulated samples and 125 intermediary samples**.** Log-rank *p*-value of up-regulated versus down-regulated is 0.09. Log-rank *p*-value of up-regulated versus intermediary is 0.27 and log-rank *p*-value of down-regulated vs intermediary is 0.056. Next, we analyzed the TCGA expression data to test whether *HIPK2* expression in GBM also correlate with GBM survival. *HIPK2* expression can be estimated by two Affymetrix probes (213763_at and 219028_at) on the chip. As expected, *HIPK2* mean expression intensity is lower in short survival patients compared to long survival patients by 1.5 for 213763_at and 1.4 for 213763_at *HIPK2* Affymetrix probes (Fig. 5c). Student T-test p value: 0.009 and 0.0002, respectively. Kaplan–Meier survival curve overlapping TCGA study core for 210 samples also illustrates shorter survival correlated to low *HIPK2* expression (Fig. 5d). In these samples there were 23 up-regulated, 25 down-regulated and 165 patients with intermediary *HIPK2* expression. The significance of difference between survival groups of samples was calculated. The log-rank *p*-value is as follow: up-regulated versus down-regulated is 0.0015, up-regulated versus intermediary is 0.2653, down-regulated vs. intermediary is 0.0025. We determined the value of correlation coefficient between *MT* probes and *HIPK2* probe. For visual display of the correlation coefficients between *MTs* and *HIPK2* pairs of probes, we formed ‘heatmap’ illustrated in Fig. 6. In this diagram the color in each cell represents the value of the correlation coefficient between the values shown by a particular pair of probes. Red color represents highly positive correlation of expression and blue represents negative correlation. The values on the main diagonal are all 1, since the correlation coefficient for a value matched with itself is 1. The value of the correlation coefficient between all *MT*s is strongly positive. On the other hand all 12 *MT* probe expression were found to be negatively correlated to *HIPK2* probe expression in these patients (R = -0.2, *p* = 0.002).

Since low *HIPK2* and high *MT*s expression were found to be associated with lower survival in GBM patients, we checked the number of samples with low *HIPK2* and high *MT* expression in TCGA cohort. 71 out of 210 (34 %) samples had this pattern of expression. In these GBM samples, *MT* associated zinc depletion may enforce inactive p53 conformation and thus may lead to decreased response to chemotherapy. Interestingly, in addition to the expected high expressed *MT*s in low *HIPK2* expressed GBM samples (71), there were also 35 (16 %) GBM samples that expressed high amounts of *MT*s and high amounts of *HIPK2*. Thus in GBM, *MT*s may be highly expressed irrespective to *HIPK2* level. These patients may also exhibit zinc depletion.

## Discussion

Our understanding of GBM tumors has expanded significantly recently. Outcomes and patient prognosis rely on histological classification combined with information on patient age and tumor size and location. However, high heterogeneity of GBM tumors among patients makes the prognosis prediction very difficult. Therefore, more individualized biomarkers may lead to better prognosis prediction and the development of novel specific targeted therapy. In search for such biomarker we analyzed 67 GBM clinical tumor samples and revealed that high *MT*s levels are associated with poor survival whereas low levels are associated to relatively good survival. We validated the results of the discovery cohort by analysis of expression data of 210 GBM patients from the TCGA database. We show here that a subset of GBM patients with high levels of *MT*s have decreased survival. Likewise, poor survival is observed in GBM patients with low levels of *HIPK2*. These observations were not always concurrent in the same patients. In fact, there are GBM patients with high *MT*s and high *HIPK2*. Thus, in addition to the established correlation between *MT* expression and *HIPK2* we suggest that other regulation mechanisms, such as epigenetic methylation [29] may increase *MT* expression even when *HIPK2* level is normal. Hence, *HIPK2* and *MT* levels (as a whole or even one or two sub isoforms such as *MT3/MT2A*) may serve as survival predictors independently. *MT*s may affect GBM in many ways: 1) High levels of *MT*s may bind alkylating agents and confer resistance to chemotherapy 2) *MT*s bind zinc and switch *p53* folding to the inactive form and thus prevent chemotherapy-activated *p53* mediated apoptosis. 3) *MT* in its apo-form may interact with *p53* and prevent inflection of gene transcription and apoptosis by *P53*. 4) *MT*s may add to the infiltrative feature of gliomas by enhancing migration, as has been shown with *MT1E* [19]. In addition, U87 GBM cell line may serve as a model for inactive *p53* conformation without mutation. In these cells we were able to rescue *p53* conformation to an active form by zinc. Thus, GBM patients exert several modes of *p53* inactivation which has survival indication and can be targeted by precise treatment. *MT*s expression may serve as a novel individualized prognostic indicator for prediction of GBM overall survival and also serve as treatment target. Another novel finding of our study is that subsets of patients with WT *p53* express high levels of *MT*s resulting in low levels of zinc and inactive *p53* even if there is no mutation in *p53*. The high *MT*s in these patients may deplete intracellular Zn levels and also interact with alkylating agents (chemotherapy) thus lowering response to treatment. In this setting the prognosis is poor and these GBM patients are expected to have short survival.

## Conclusions

Our study demonstrates that high metallothionein levels in GBM patients are associated with poor survival. Therefore, *MT* expression may be a potential novel prognostic biomarker for GBM. In addition, we have discovered that the *p53* conformation in GBM U87 cell line is inactive and can be altered to the active form by addition of zinc. Thus GBM U87 cell line may be a good model for patients with non active WT *p53* resulting from high levels of *MTs* without mutations in the *p53* gene.

### Availability of supporting data

The TCGA GBM samples Affymetrix Affy HT Human Genome U133 expression data are publicly available at: TCGA data portal (https://tcga-data.nci.nih.gov/tcga).

## Abbreviations

GBM: Glioblastoma multiforme
MT: Metallothionein
NHA: Normal human astrocytes
RT-PCR: Real-time polymerase chain reaction
RQ: Relative quantification
WHO: World health organization
ZnCL2: Zinc chloride

## References

[1] Furnari FB, Fenton T, Bachoo RM, Mukasa A, Stommel JM, Stegh A, Hahn WC, Ligon KL, Louis DN, Brennan C (2007). Malignant astrocytic glioma: genetics, biology, and paths to treatment. Genes Dev.

[2] Li S, Li L, Zhu Y, Huang C, Qin Y, Liu H, et al. Coexistence of EGFR with KRAS, or BRAF, or PIK3CA somatic mutations in lung cancer: a comprehensive mutation profiling from 5125 Chinese cohorts. Br J Cancer. 2014 May 27;110(11):2812–20. doi: 10.1038/bjc.2014.210. Epub 2014 Apr 17. 10.1038/bjc.2014.210 PMC4037826 24743704

[3] Chaudhry NS, Shah AH, Ferraro N, Snelling BM, Bregy A, Madhavan K, Komotar RJ (2013). Predictors of long-term survival in patients with glioblastoma multiforme: advancements from the last quarter century. Cancer Investig.

[4] Brower JV, Clark PA, Lyon W, Kuo JS. MicroRNAs in cancer: Glioblastoma and glioblastoma cancer stem cells. Neurochem Int. 2014. 10.1016/j.neuint.2014.06.002 PMC4390175 24937770

[5] Chiang JH, Cheng WS, Hood L, Tian Q (2014). An epigenetic biomarker panel for glioblastoma multiforme personalized medicine through DNA methylation analysis of human embryonic stem cell-like signature. Omics J Integ Biol.

[6] Olar A, Aldape KD (2012). Biomarkers classification and therapeutic decision-making for malignant gliomas. Curr Treat Options in Oncol.

[7] Jain R, Poisson LM, Gutman D, Scarpace L, Hwang SN, Holder CA, Wintermark M, Rao A, Colen RR, Kirby J (2014). Outcome prediction in patients with glioblastoma by using imaging, clinical, and genomic biomarkers: focus on the nonenhancing component of the tumor. Radiology.

[8] Miles AT, Hawksworth GM, Beattie JH, Rodilla V (2000). Induction, regulation, degradation, and biological significance of mammalian metallothioneins. Crit Rev Biochem Mol Biol.

[9] Stennard FA, Holloway AF, Hamilton J, West AK (1994). Characterisation of six additional human metallothionein genes. Biochim Biophys Acta.

[10] Palmiter RD, Findley SD, Whitmore TE, Durnam DM (1992). MT-III, a brain-specific member of the metallothionein gene family. Proc Natl Acad Sci U S A.

[11] Quaife CJ, Findley SD, Erickson JC, Froelick GJ, Kelly EJ, Zambrowicz BP, Palmiter RD (1994). Induction of a new metallothionein isoform (MT-IV) occurs during differentiation of stratified squamous epithelia. Biochemistry.

[12] Dunn MA, Blalock TL, Cousins RJ (1987). Metallothionein. Proc Soc Exp Biol Med.

[13] Puca R, Nardinocchi L, Bossi G, Sacchi A, Rechavi G, Givol D, D’Orazi G (2009). Restoring wtp53 activity in HIPK2 depleted MCF7 cells by modulating metallothionein and zinc. Exp Cell Res.

[14] Habel N, Hamidouche Z, Girault I, Patino-Garcia A, Lecanda F, Marie PJ, Fromigue O (2013). Zinc chelation: a metallothionein 2A’s mechanism of action involved in osteosarcoma cell death and chemotherapy resistance. Cell Death Dis.

[15] Falnoga I, Zelenik Pevec A, Slejkovec Z, Znidaric MT, Zajc I, Mlakar SJ, Marc J (2012). Arsenic trioxide (ATO) influences the gene expression of metallothioneins in human glioblastoma cells. Biol Trace Elem Res.

[16] Petering DH, Zhu J, Krezoski S, Meeusen J, Kiekenbush C, Krull S, Specher T, Dughish M (2006). Apo-metallothionein emerging as a major player in the cellular activities of metallothionein. Exp Biol Med (Maywood).

[17] Ostrakhovitch EA, Olsson PE, Jiang S, Cherian MG (2006). Interaction of metallothionein with tumor suppressor p53 protein. FEBS Lett.

[18] Xia N, Liu L, Yi X, Wang J (2009). Studies of interaction of tumor suppressor p53 with apo-MT using surface plasmon resonance. Anal Bioanal Chem.

[19] Ryu HH, Jung S, Jung TY, Moon KS, Kim IY, Jeong YI, Jin SG, Pei J, Wen M, Jang WY (2012). Role of metallothionein 1E in the migration and invasion of human glioma cell lines. Int J Oncol.

[20] Puca R, Nardinocchi L, Gal H, Rechavi G, Amariglio N, Domany E, Notterman DA, Scarsella M, Leonetti C, Sacchi A (2008). Reversible dysfunction of wild-type p53 following homeodomain-interacting protein kinase-2 knockdown. Cancer Res.

[21] Margalit O, Simon AJ, Yakubov E, Puca R, Yosepovich A, Avivi C, Jacob-Hirsch J, Gelernter I, Harmelin A, Barshack I (2012). Zinc supplementation augments in vivo antitumor effect of chemotherapy by restoring p53 function. Int J Cancer J Int Cancer.

[22] Puca R, Nardinocchi L, Givol D, D’Orazi G (2010). Regulation of p53 activity by HIPK2: molecular mechanisms and therapeutical implications in human cancer cells. Oncogene.

[23] Puca R, Nardinocchi L, Porru M, Simon AJ, Rechavi G, Leonetti C, Givol D, D’Orazi G (2011). Restoring p53 active conformation by zinc increases the response of mutant p53 tumor cells to anticancer drugs. Cell Cycle.

[24] Comprehensive genomic characterization defines human glioblastoma genes and core pathways.Cancer Genome Atlas Research Network. Nature. 2008;455(7216):1061–1068. 10.1038/nature07385 PMC2671642 18772890

[25] Ory K, Legros Y, Auguin C, Soussi T (1994). Analysis of the most representative tumour-derived p53 mutants reveals that changes in protein conformation are not correlated with loss of transactivation or inhibition of cell proliferation. EMBO J.

[26] Foster BA, Coffey HA, Morin MJ, Rastinejad F (1999). Pharmacological rescue of mutant p53 conformation and function. Science.

[27] Joerger AC, Fersht AR (2007). Structure-function-rescue: the diverse nature of common p53 cancer mutants. Oncogene.

[28] Yu X, Vazquez A, Levine AJ, Carpizo DR (2012). Allele-specific p53 mutant reactivation. Cancer Cell.

[29] Majumder S, Kutay H, Datta J, Summers D, Jacob ST, Ghoshal K (2006). Epigenetic regulation of metallothionein-i gene expression: differential regulation of methylated and unmethylated promoters by DNA methyltransferases and methyl CpG binding proteins. J Cell Biochem.

